# Association Between Dietary Adherence and Cognitive Function Among Rural Older Patients with Cardiometabolic Multimorbidity: The Moderating Role of Health Management

**DOI:** 10.3390/nu17243820

**Published:** 2025-12-06

**Authors:** Fangfang Hu, Lingzhong Xu, Wenzhe Qin

**Affiliations:** 1 Department of Social Medicine and Health Management, School of Public Health, Cheeloo College of Medicine, Shandong University, Jinan 250012, China; hufang@mail.sdu.edu.cn (F.H.); lzxu@sdu.edu.cn (L.X.); 2 National Health Commission (NHC) Key Laboratory of Health Economics and Policy Research, Shandong University, Jinan 250012, China

**Keywords:** dietary adherence, cognitive function, health management, rural older patients, cardiometabolic multimorbidity

## Abstract

**Background:** Cardiometabolic multimorbidity (CMM) among rural older adults is becoming increasingly prevalent. Although previous studies have confirmed that dietary adherence significantly affects cognitive function, the mechanisms underlying this relationship remain insufficiently explored. This cross-sectional study aimed to examine the association between dietary adherence and cognitive function among rural older patients with CMM and further explored the moderating role of health management. **Methods:** The sample was selected using a multi-stage cluster random sampling method and a total of 1487 rural older patients with CMM were ultimately included. Hierarchical regression analysis was employed to examine the association between dietary adherence and cognitive function and test the moderating role of health management. Simple slope analysis was further employed to explore the moderation effect. **Results:** The cognitive function and dietary adherence scores of rural older patients with CMM were 20.790 ± 6.278 and 2.157 ± 0.286, respectively. Dietary adherence was significantly and positively associated with cognitive function (β = 2.000, *p* < 0.001) and health management moderated this relationship (β = 4.287, *p* = 0.009). Compared with rural older patients with CMM who did not receive health management, the positive predictive effect of dietary adherence on cognitive function was significantly stronger among those who received health management. **Conclusions:** Higher dietary adherence was positively associated with better cognitive function among rural older patients with CMM, and receiving health management further strengthened this association. These findings emphasized the importance of integrating dietary guidance within community-based health management interventions to promote cognitive function.

## 1. Introduction

With the acceleration of population aging in China, multimorbidity is becoming increasingly prevalent among the elderly population [1]. Cardiometabolic multimorbidity (CMM) refers to a situation where a person has two or more cardiometabolic diseases (CMDs) simultaneously [2]. This is a common pattern of comorbidity aggregation among the elderly population [3]. The number of patients with CMM in rural China continues to increase [4]. CMM is associated with various adverse health outcomes, including cognitive decline [5], worsening of depressive symptoms [6], and increased risk of dementia [7], and even leads to increased risks of disability and death [8], imposing a heavy burden on patients, families, and society, and becoming one of the main obstacles to healthy aging [9,10].

Cognitive function refers to the mental processes through which the human brain receives and processes external information, thereby acquiring and applying knowledge. It encompasses multiple cognitive domains, including memory, orientation, attention, language, executive function, and perception [11]. Cognitive impairment arises when one or more of these domains are damaged and is a common neurodegenerative condition among older adults. Cognitive impairment includes mild cognitive impairment and dementia. Its early manifestations typically involve significant memory decline accompanied by reduced ability to perform daily and social activities, while in the later stages, behavioral disturbances and psychiatric symptoms may emerge, ultimately leading to a complete loss of independent living ability [12]. The integrity of cognitive function gradually declines with age, and the prevalence of cognitive impairment and multimorbidity among older adults in China has shown a marked upward trend. Studies have indicated that the coexistence of multiple chronic diseases accelerates the deterioration of cognitive function—the greater the number of chronic conditions, the more severe the cognitive decline [13,14,15]. Moreover, the incidence of cognitive impairment increases significantly with age. Dove et al. found that cognitive decline accelerates in a dose-dependent manner as the number of cardiometabolic comorbidities increases [16]. Cognitive impairment among older adults has become a major public health challenge in China, placing significant strain on the healthcare and long-term care systems. The prevention and management of cognitive impairment have thus become critical national strategies for addressing population aging. However, there is still a lack of effective medications for preventing or treating cognitive decline. Therefore, understanding the current status of cognitive function among older patients with CMM, with an emphasis on the early prevention and management of cognitive impairment, has become a major research focus [17]. This is crucial for preventing or delaying cognitive decline, improving overall quality of life, and effectively addressing the challenges posed by an aging society.

Among various modifiable lifestyle factors, dietary behavior has attracted increasing attention for its direct effects on cognitive function through mechanisms such as antioxidant activity, neurotransmitter regulation, and the maintenance of brain structure [18]. Healthy dietary patterns, such as the Mediterranean diet and the DASH diet, have been shown to delay cognitive decline by improving vascular function, reducing oxidative stress and neuroinflammation, and maintaining metabolic homeostasis [19,20,21]. Furthermore, the ketogenic diet also has a positive effect on cognitive function [22]. In contrast, unhealthy dietary habits are strongly associated with cognitive impairment [4,23]. The recommended dietary guidelines for older patients with CMM emphasize the intake of vegetables, fruits, legumes, whole grains, nuts, and plant-based oils, while allowing limited or moderate consumption of animal-based foods, added sugars, and saturated fats [24]. Dietary adherence refers to the degree to which an individual’s diet behaviors align with medical recommendations. It is an important indicator for evaluating the effectiveness of health management among older patients with CMM. In populations with multimorbidity, dietary adherence exerts an even more significant influence on disease progression and quality of life [25,26]. These pathways may be particularly relevant for rural older patients with CMM in China. This population often faces long-term exposure to high-salt, high-carbohydrate, and low-diversity diets due to traditional eating habits and limited food accessibility, potentially amplifying diet-related cognitive risks. The effects of dietary behavior may be particularly salient in rural older adults who often have limited access to consistent healthcare, potentially exacerbating underlying unfavorable health outcomes. However, most existing studies focus on single diseases or healthy populations. For rural older patients with CMM in China, the relationship between dietary adherence and cognitive function remains underexplored. Investigating this association can help elucidate modifiable behavioral mechanisms underlying cognitive decline and provide scientific evidence for health promotion among rural older adults. Given this, we proposed the first hypothesis: dietary adherence positively predicts cognitive function among rural older patients with CMM.

Meanwhile, health management represents a crucial approach to improving disease control and behavioral adherence among patients with chronic conditions. Community-based health management services for older adults can enhance health awareness and self-management capacity, thereby improving dietary behaviors and supporting cognitive function [27,28]. Previous studies have demonstrated that older adults receiving systematic health management show better disease knowledge, behavioral adherence, and psychological well-being compared with those without such services [29,30,31]. Comprehensive health management has also been shown to slow the progression of chronic disease, reduce hospital readmissions, and improve quality of life. Despite the established link between diet and cognition, it remains unclear whether existing health management services in rural older adults can potentiate this relationship, representing a critical gap in knowledge. Clarifying this moderating effect is essential, as it could reveal how existing health management services might be leveraged to amplify the cognitive benefits of dietary interventions. Therefore, we proposed the second hypothesis: health management plays a moderating role between dietary adherence and cognitive function among rural older patients with CMM.

**Figure 1:**
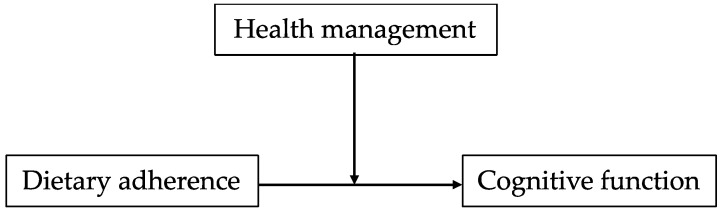
Theoretical model.

Based on the above hypotheses, the theoretical model of “dietary adherence—health management—cognitive function” constructed in this study was shown in Figure 1. This study aimed to: (1) describe the current status of dietary adherence and cognitive function among rural older patients with CMM; (2) examine the association between dietary adherence and cognitive function; and (3) explore the moderating role of health management in this association. Through empirical analysis, this study aims to identify modifiable behavioral factors associated with cognitive function, clarify the role of health management in promoting healthy behaviors and cognitive function, and provide both theoretical and practical implications for optimizing chronic disease management and advancing healthy aging policies in rural China.

## 2. Materials and Methods

### 2.1. Study Design and Sample

This cross-sectional study recruited participants using a multi-stage cluster random sampling method. Detailed methods were described elsewhere [32]. First, based on the level of socioeconomic development (measured by per capita gross domestic product, GDP) and geographical location, one prefecture-level city was selected from each of the eastern (Weifang City), central (Tai’an City), and western (Dezhou City) regions of Shandong Province, aiming to ensure the representativeness of the sample in terms of regional distribution and economic development levels. Next, one county-level city was randomly selected from each prefecture-level city. These were Gaomi City, Xintai City, and Laoling City, respectively. Then, a probability proportional to size (PPS) sampling method was used to select three to four townships or sub-districts from each county-level city, including four from Gaomi City, four from Xintai City, and three from Laoling City, totaling 11 townships/sub-districts. Subsequently, six villages were randomly selected from each township or sub-district, totaling 66 villages. Finally, in each sample village, based on the health records provided by the local village health center or community health service station, older patients with CMM meeting the inclusion criteria were selected as the initial sample frame. Subsequently, 20 eligible older patients with CMM were randomly selected as the survey subjects for on-site investigation. This plan aimed to investigate 1320 rural older patients with CMM, and 1505 were actually surveyed. Eventually, 1487 valid questionnaires were recovered, with an effective questionnaire recovery rate of 98.80%. The final sample of this study consisted of 1487 rural older patients with CMM.

### 2.2. Cognitive Function Assessment

The Mini-Mental State Examination (MMSE) was used to assess the cognitive function of rural older patients with CMM. This scale was developed by Folstein et al. in 1975 and is a simple and feasible cognitive function assessment tool [33]. It involves seven aspects: time orientation, place orientation, immediate memory, attention and calculation ability, delayed memory, language ability, and visuospatial ability. It consists of 30 items, with 1 point awarded for each correct answer and 0 points for incorrect or unknown answers. The total score ranges from 0 to 30, with higher scores indicating better cognitive function. Cognitive impairment was defined using education-adjusted cut-off scores as follows: illiterate (≤17), primary school (≤20), junior high school (≤22), and high school or above (≤24). In this study, the Cronbach’s α coefficient of the MMSE was 0.909.

### 2.3. Dietary Adherence Assessment

This study, with reference to the EAT-Lancet dietary pattern, developed and weighted a dietary adherence assessment questionnaire suitable for rural older adults with CMM [24]. The authority coefficients (Cr) of the two rounds of expert consultation were both ≥0.850, and Kendall’s W coefficients were 0.168 and 0.188, respectively (both *p* < 0.001), indicating good consistency in expert opinions. The questionnaire consists of 16 items, covering three dimensions: healthy food intake (such as vegetables, fruits, fish, beans, etc.), limiting intake of unhealthy foods (such as red meat, sweets/sugary beverages, deep-fried foods, high-salt foods, etc.), and cooking and staple food choices (such as the use of vegetable oil, control of refined rice and flour). Each item is scored based on the frequency of intake or occurrence of behavior. Items such as red meat, sweets/sugary beverages, deep-fried foods, high-salt foods, and spicy foods were reverse-coded due to their adverse health implications. The full dietary adherence assessment questionnaire is provided in the Supplementary Materials (Table S1). The normalized weights were calculated through the Analytic Hierarchy Process (AHP) method, with weight coefficient ranges from 0.045 to 0.093. After weighting, the total score of dietary adherence ranged from 0 to 3.649, which can be directly used for the quantitative evaluation of dietary adherence among rural older patients with CMM. The higher the score, the better the dietary adherence.

### 2.4. Health Management Assessment

The utilization of health management services among rural older patients with CMM was assessed using a single self-reported question: “During the past 12 months, have you received any health management services provided by your family doctor team (e.g., health guidance, chronic disease follow-up management)?” Responses were coded as a binary variable, where 0 indicated “never received” and 1 indicated “received”. While this binary measure offers limited granularity regarding the frequency, type, or quality of services, it was selected to align with the study’s primary focus on dietary adherence and to reduce participant burden in rural older patients with CMM.

### 2.5. Covariates

Covariates were selected a priori based on their established association with cognitive function in the literature and included gender (male, female); age group (60~69, 70~79, ≥80); education level (illiteracy, primary school, middle school and above); marital status (married, unmarried/divorced/widowed); living arrangement (living alone, living only with spouse, living with spouse and children); personal annual income (5000, 5000~10,000, ≥10,000); medical insurance type (Basic Medical Insurance for Urban Employee (UEBMI), Basic Medical Insurance for Urban and Rural Residents (RBMI), Others); self-rated health (Poor, General, Good); number of CMDs (2, 3, ≥4); smoke (current smoker, former smoker, non-smoker); and alcohol (current drinker, former drinker, non-drinker), following the methodology in previous studies.

### 2.6. Statistical Analysis

The database was established using EpiData 3.1 software, and data cleaning and statistical analyses were conducted with Stata/SE (version 15.1; Stata Corp, College Station, TX, USA). Categorical variables were described using frequencies and percentages, while continuous variables were presented as the mean ± standard deviation (M ± SD). Pearson correlation analysis was employed to examine the relationships among the main variables. Independent-sample *t*-tests were conducted to compare the mean differences in dietary adherence and cognitive function across different health management statuses. Hierarchical regression analysis was further performed to explore the association between dietary adherence and cognitive function, as well as the moderating effect of health management. Hierarchical regression was used to assess the incremental contribution of dietary adherence and interaction effects beyond covariates. Prior to creating interaction terms, all continuous predictor variables were mean-centered to mitigate multicollinearity and facilitate the interpretation of coefficients. Multicollinearity was assessed using variance inflation factors (VIF), with all values below 10 indicating no serious multicollinearity. In addition, simple slope plots were generated to visually illustrate the direction and strength of the moderating effect of health management on their relationship. All statistical tests were two-tailed, and a *p*-value < 0.05 was considered statistically significant.

## 3. Results

### 3.1. Characteristics of Study Participants

A total of 1487 rural older patients with CMM were included in this study, with a mean age of 71.32 ± 5.86 years. Among them, 519 were male (34.90%) and 968 were female (65.10%). Regarding age distribution, 574 patients (38.60%) were aged 60–69 years, 789 (53.06%) were aged 70–79 years, and 124 (8.34%) were aged 80 years or above. In terms of education level, 691 patients (46.47%) were illiterate, 491 (33.02%) had primary education, and 305 (20.51%) had junior high school education and above. A total of 1103 patients (74.18%) were married, while 384 (25.82%) were unmarried/divorced/widowed. Regarding living arrangements, 302 (20.31%) lived alone, 977 (65.70%) lived only with their spouse, and 208 (13.99%) lived with their spouse and children. The majority of patients (59.18%) had a personal annual income of less than 5000 yuan. Most participants (98.10%) were covered by RBMI. As for self-rated health status, 385 patients (25.89%) rated their health as poor, 530 (35.64%) as general, and 572 (38.47%) as good. Regarding the number of CMDs, 818 (55.01%) had two types and 462 (31.06%) had three types. During the past year, 1342 patients (90.25%) had received health management services. In terms of lifestyle behaviors, 1135 (76.32%) had never smoked, and 1112 (74.78%) had never consumed alcohol. Details are shown in Table 1.

### 3.2. Common Method Bias Test

The control of common method bias can be divided into procedural control and statistical control. In terms of procedural control, we emphasized anonymity and confidentiality in the questionnaire survey and included several attention-check items, which can effectively mitigate the potential for common method bias to some extent. For statistical control, we conducted Harman’s single-factor test, and the results showed that the first common factor accounted for only 18.813% of the variance, which is below the critical threshold of 40%, indicating that severe common method bias is unlikely in this study.

### 3.3. Descriptive Statistics and Correlation Analysis of the Main Variables

Table 2 presents the means, standard deviations, and correlation coefficients of the main variables. The dietary adherence score was 2.157 ± 0.286, health management score was 1.900 ± 0.297, and cognitive function score was 20.790 ± 6.278. Dietary adherence was significantly and positively correlated with health management (*r* = 0.081, *p* < 0.01) and cognitive function (*r* = 0.140, *p* < 0.001). In addition, there was a significant positive correlation between health management and cognitive function (*r* = 0.054, *p* < 0.05).

### 3.4. Difference Analysis of Dietary Adherence and Cognitive Function

As shown in Table 3, the results of the *t*-test indicated that both dietary adherence and cognitive function differed significantly across health management status groups (*p* < 0.05). Older patients with CMM who received health management demonstrated significantly higher levels of dietary adherence and cognitive function compared with those who did not receive health management.

### 3.5. Hierarchical Regression Analysis of Cognitive Function

Hierarchical regression analysis was conducted to examine the relationship between dietary adherence and cognitive function, as well as the moderating effect of health management. In the regression analysis, control variables were entered in the first step, dietary adherence and health management were entered in the second step, and the interaction term between dietary adherence and health management was entered in the third step. The results of the hierarchical regression analysis are shown in Table 4. After controlling for age, gender, education level, marital status, living arrangement, personal annual income, medical insurance type, self-rated health, number of CMDs, smoke, alcohol, dietary adherence was found to significantly and positively predict cognitive function (β = 2.000, *p* < 0.001). Health management did not have a significant predictive effect on cognitive function (β = 0.515, *p* > 0.05). However, the interaction term between dietary adherence and health management significantly predicted cognitive function (β = 4.287, *p* = 0.009), indicating that health management moderated the relationship between dietary adherence and cognitive function.

**Figure 2:**
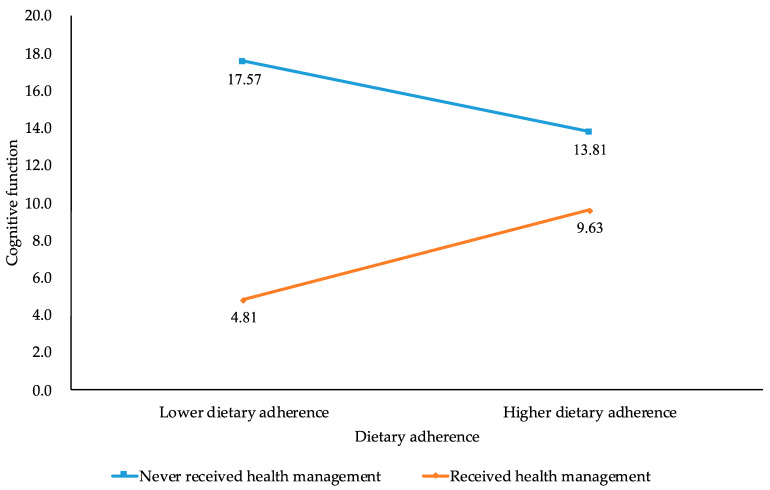
The moderating effect of health management on the relationship between dietary adherence and cognitive function.

Simple slope analysis was conducted to examine the moderating effect of health management [34]. As shown in Figure 2, dietary adherence did not significantly predict cognitive function in older patients without health management (β = −1.878, *p* > 0.05, 95%CI [−5.112, 1.064]). In contrast, among those receiving health management, dietary adherence positively predicted cognitive function (β = 2.409, *p* < 0.001, 95%CI [1.485, 3.482]), with each one-unit increase in dietary adherence associated with an average 2.409-point increase in cognitive scores. These findings indicate that health management enhances the positive effect of dietary adherence on cognitive function.

## 4. Discussion

In this study, cognitive function score among rural older patients with CMM was generally at a moderate level, suggesting that some patients had already developed mild cognitive impairment. Compared with previous studies on the general older population, the cognitive function scores in this sample were lower [35,36], which may be attributed to accelerated cognitive decline associated with CMM, potentially mediated by chronic inflammation, oxidative stress, and vascular dysfunction. Rural older patients with CMM generally have the problem of poor dietary adherence. Some studies have pointed out that 60% of the total score of the scale can be regarded as a medium level of adherence [37]. In this study, the total score of dietary adherence was 3.649, indicating that the dietary adherence of the rural older patients with CMM was at a medium to low level. Many rural older adults demonstrated difficulties in maintaining balanced diets, mainly due to traditional high-salt, high-oil eating habits, limited nutrition literacy, and constrained economic conditions [38]. Moreover, the low participation rate in structured health management programs further reduces opportunities for professional dietary guidance [39].

This study found that dietary adherence was significantly and positively associated with cognitive function among rural older patients with CMM. This finding was consistent with previous studies showing that better dietary adherence is linked to higher cognitive scores among older adults [40,41]. A healthy dietary pattern, which is characterized by a high consumption of fruits, vegetables, and fish, and low intake of saturated fat, sugar, and salt, has been shown to help prevent cognitive decline through multiple mechanisms, including improved vascular health, reduced systemic inflammation, and maintenance of metabolic and neuronal stability [20,42]. Specifically, carotenoids, vitamin E, and vitamin C act as potent antioxidants that eliminate excessive reactive oxygen species in brain tissue, thereby reducing oxidative stress-induced neuronal damage. Meanwhile, vitamins B6 and D exert neuroprotective effects by participating in neurotransmitter synthesis and maintaining the integrity of the blood–brain barrier [43]. In contrast, poor dietary adherence may exacerbate oxidative stress and insulin resistance, thereby accelerating neurodegenerative processes [23]. In the context of multimorbidity, a healthy diet not only helps control diseases but also enhances cognitive resilience. However, among rural populations, practical barriers such as limited food diversity and cultural dietary preferences often hinder optimal adherence. Therefore, rural health policies should focus on nutrition education, incorporate nutritionists into rural medical teams to provide professional dietary guidance and chronic disease management services, and promote culturally adapted interventions to improve dietary quality and cognitive outcomes.

The key finding of this study was that health management strengthened the positive association between dietary adherence and cognitive function, indicating that structured health management services can enhance the cognitive benefits of better dietary adherence [44,45]. The preliminary nature of this finding is due to the single-item measurement approach. Health management services, including regular follow-ups, health education, and personalized dietary counseling, can enhance patients’ self-efficacy and promote sustained behavioral changes, thereby consolidating the cognitive benefits of a healthy diet [27]. Regular follow-ups and health status monitoring may help patients adjust unhealthy behaviors in a timely manner, thus improving the effectiveness of interventions. Personalized dietary or lifestyle advice provided by primary healthcare professionals may enhance the influence of dietary adherence on maintaining cognitive function. Furthermore, health management may also help maintain cognitive function by improving adherence to medication and lifestyle modifications and facilitating the early detection and control of multimorbidity conditions [46]. This moderating effect supports the concept of behavior–environment synergy, where individual-level health behaviors (dietary adherence) and system-level support (health management) interact to produce optimal cognitive outcomes. It also emphasizes the crucial role of primary care and community health services in promoting healthy aging, particularly in rural areas with limited personal resources and healthcare accessibility [47]. Therefore, policymakers should prioritize expanding the coverage and quality of rural health management services, integrating dietary guidance and cognitive screening into primary care. In addition, strengthening family doctor teams is recommended to promote sustained and effective health management among rural older patients with CMM.

The strengths of this study include a large sample of rural older patients with CMM and the application of a validated measure of dietary adherence. To the best of our knowledge, this is the first study in China to preliminary investigate the association between dietary adherence and cognitive function among older patients with CMM, as well as the moderating role of health management. However, several limitations should be acknowledged. First, the cross-sectional design precludes causal inference between dietary adherence and cognitive function. Individuals with better cognitive function may also be more likely to adhere to dietary guidance or engage in health management, which could potentially influence the observed associations. This possibility limits causal inference and should be further explored in future longitudinal studies. Second, dietary adherence and health management were assessed through self-reported questionnaires, which may be subject to recall bias and social desirability bias. Future research could incorporate objective biomarkers or dietary records. Third, the binary measure of health management may not capture the heterogeneity in service quality, frequency, and intensity. A key limitation of this study is the significant imbalance in group sizes, with approximately 90% of participants receiving health management. This imbalance may have influenced the statistical power and the generalizability of the between-group comparisons. Future studies should further differentiate types and intensities of health management interventions. Despite these limitations, this study provides valuable insights into the individual behavioral and environmental determinants of cognitive function among rural older patients with CMM.

## 5. Conclusions

In conclusion, this study demonstrated a significant positive association between higher dietary adherence and better cognitive function among rural older patients with CMM, with health management status moderating this association. These findings highlight the importance of both individual dietary behavior and health management in maintaining cognitive function health in later life. The observed associations support the potential value of implementing comprehensive, community-based health management programs that incorporate dietary guidance as part of multidimensional strategies for cognitive health maintenance in rural older patients with CMM.

## Figures and Tables

**Table 1:** Demographic characteristics of rural older patients with CMM.

Characteristics	Group	*N*	%
Gender			
	Male	519	34.90
	Female	968	65.10
Age group			
	60~69	574	38.60
	70~79	789	53.06
	≥80	124	8.34
Educational level			
	Illiteracy	691	46.47
	Primary school	491	33.02
	Junior school and above	305	20.51
Marital status			
	Unmarried/divorced/widowed	384	25.82
	Married	1103	74.18
Living arrangement			
	Living alone	302	20.31
	living only with spouse	977	65.70
	Living with spouse and children	208	13.99
Personal annual income (CNY)			
	<5000	880	59.18
	5000~10,000	368	24.75
	≥10,000	239	16.07
Medical insurance type			
	UEBMI	38	2.56
	RBMI	1429	98.10
	Others	20	1.34
Self-rated health			
	Poor	385	25.89
	General	530	35.64
	Good	572	38.47
Number of CMDs			
	2	818	55.01
	3	462	31.06
	≥4	207	13.93
Health management			
	Never received	145	9.75
	Received	1342	90.25
Smoke			
	Current smoker	183	12.31
	Former smoker	169	11.37
	Non-smoker	1135	76.32
Alcohol			
	Current drinker	235	15.80
	Former drinker	140	9.42
	Non-drinker	1112	74.78

Note: UEBMI, Basic Medical Insurance for Urban Employee; RBMI, Basic Medical Insurance for Urban and Rural Residents; CMDs, cardiometabolic diseases.

**Table 2:** Descriptive statistics and correlation analysis of the main variables.

Characteristics	M ± SD	1	2	3
1. Dietary adherence	2.157 ± 0.286	1		
2. Health management	1.900 ± 0.297	0.081 **	1	
3. Cognitive function	20.790 ± 6.278	0.140 ***	0.054 *	1

Note: *** *p* < 0.001, ** *p* < 0.01, * *p* < 0.05.

**Table 3:** Difference analysis of dietary adherence and cognitive function.

Characteristics	Health Management		*t*	*p*
	Never Received	Received		
Dietary adherence	2.089 ± 0.272	2.166 ± 0.281	−3.149	0.002
Cognitive function	19.760 ± 6.366	20.900 ± 6.260	−2.087	0.037

**Table 4:** Hierarchical regression analysis of cognitive function among rural older patients with CMM.

Characteristics		Model 1			Model 2			Model 3		
		β	*p*	95%CI	β	*p*	95%CI	β	*p*	95%CI
Gender (ref: Male)										
Female		−0.430	0.292	[−1.231, 0.371]	−0.519	0.202	[−1.316, 0.279]	−0.567	0.163	[−1.364, 0.229]
Age group (ref: 60~69)										
70~79		−0.739	0.012	[−1.314, −0.164]	−0.803	0.006	[−1.375, −0.230]	−0.85	0.004	[−1.422, −0.277]
≥80		−2.662	<0.001	[−3.689, −1.634]	−2.820	<0.001	[−3.843, −1.796]	−2.817	<0.001	[−3.839, −1.796]
Educational level (ref: Illiteracy)										
Primary school		5.035	<0.001	[4.417, 5.653]	4.966	<0.001	[4.351, 5.582]	4.954	<0.001	[4.340, 5.568]
Junior school and above		7.164	<0.001	[6.346, 7.982]	6.951	<0.001	[6.132, 7.770]	6.897	<0.001	[6.079, 7.715]
Marital status (ref: Unmarried/divorced/widowed)										
Married		0.330	0.564	[−0.792, 1.451]	0.409	0.473	[−0.707, 1.524]	0.357	0.529	[−0.757, 1.471]
Living arrangement (ref: Living alone)										
living only with spouse		0.241	0.701	[−0.989, 1.471]	0.097	0.877	[−1.127, 1.321]	0.121	0.846	[−1.101, 1.343]
Living with others		0.2	0.726	[−0.917, 1.316]	0.098	0.862	[−1.013, 1.209]	0.08	0.887	[−1.028, 1.189]
Personal annual income (CNY) (ref: <5000)										
5000~10,000		1.673	<0.001	[1.045, 2.300]	1.668	<0.001	[1.044, 2.291]	1.666	<0.001	[1.043, 2.288]
≥10,000		2.065	<0.001	[1.272, 2.858]	1.945	<0.001	[1.153, 2.736]	1.952	<0.001	[1.162, 2.742]
Medical insurance type (ref: UEBMI)										
RBMI		1.256	0.153	[−0.469, 2.981]	1.261	0.149	[−0.453, 2.976]	1.294	0.138	[−0.418, 3.005]
Others		−0.776	0.587	[−3.583, 2.030]	−0.541	0.704	[−3.333, 2.251]	−0.529	0.710	[−3.315, 2.258]
Self-rated health (ref: Poor)										
General		1.578	<0.001	[0.900, 2.256]	1.449	<0.001	[0.773, 2.126]	1.426	<0.001	[0.750, 2.101]
Good		1.206	0.001	[0.521, 1.891]	1.115	0.001	[0.433, 1.797]	1.087	0.002	[0.406, 1.768]
Number of CMDs (ref: 2)										
3		0.109	0.717	[−0.478, 0.696]	0.138	0.642	[−0.446, 0.722]	0.138	0.641	[−0.444, 0.721]
≥4		−0.012	0.976	[−0.814, 0.789]	−0.077	0.850	[−0.875, 0.721]	−0.103	0.800	[−0.899, 0.693]
Smoke (ref: Current smoker)										
Former smoker		0.339	0.552	[−0.778, 1.457]	0.249	0.661	[−0.864, 1.361]	0.209	0.712	[−0.901, 1.320]
Non-smoker		1.178	0.015	[0.230, 2.125]	1.049	0.030	[0.105, 1.994]	1.043	0.030	[0.100, 1.985]
Alcohol (ref: Current drinker)										
Former drinker		−0.706	0.215	[−1.822, 0.411]	−0.755	0.182	[−1.866, 0.355]	−0.751	0.184	[−1.859, 0.358]
Non-drinker		−1.087	0.015	[−1.965, −0.209]	−1.139	0.011	[−2.013, −0.266]	−1.171	0.009	[−2.043, −0.299]
Dietary adherence					2.000	<0.001	[1.060, 2.941]	−1.878	0.226	[−4.922, 1.165]
Health management (ref: Never received)										
Received					0.515	0.247	[−0.358, 1.388]	−8.468	0.014	[−15.231, −1.705]
Dietary adherence × health management (ref: Dietary adherence × never received)										
Dietary adherence × received								4.287	0.009	[1.087, 7.488]
Adjusted *R^2^*			0.3471			0.3549			0.3574	

Note: UEBMI, Basic Medical Insurance for Urban Employee; RBMI, Basic Medical Insurance for Urban and Rural Residents; CMDs, cardiometabolic diseases.

## Data Availability

The data of this study are available to researchers upon reasonable request to corresponding authors due to patient privacy.

## Supplementary Materials

The following supporting information can be downloaded at: https://www.mdpi.com/article/10.3390/nu17243820/s1, Table S1: Dietary adherence assessment questionnaire.
