# Supplementary material for: Association Between Dietary Adherence and Cognitive Function Among Rural Older Patients with Cardiometabolic Multimorbidity: The Moderating Role of Health Management
Source: Nutrients. 2025 Dec 6;17(24):3820. doi: 10.3390/nu17243820 (PMC12735681; doi:10.3390/nu17243820)
Supplement: Supplementary file 1 [file nutrients-17-03820-s001.zip › nutrients-3984295-supplementary.pdf]

Table S1 Dietary adherence assessment questionnaire

	Over the past 12 months, how often did you consume the following foods?<br>(0) Never, (1)≤ 3 times per month, (2)1–3 times per week,(3)4–6 times per week, (4)≥1 time per day<br>*(0) ≥1 time per day, (1)4–6 times per week, (2)1–3 times per week, (3)≤ 3 times per month, (4)Never	Weight coefficient
1.	Fresh vegetables	0.0766
2.	Fresh fruits	0.0627
3.	Red meat (pork, beef, lamb, etc.) *	0.0489
4.	White meat (chicken, duck, goose, etc.)	0.0537
5.	Fish or shellfish / seafood (fish, shrimp, etc.)	0.0450
6.	Legumes and soy products (soybeans, mung beans; tofu, yuba, etc.)	0.0566
7.	Eggs	0.0605
8.	Milk and dairy products (milk, yogurt, milk powder, etc.)	0.0626
9.	Nuts (peanuts, sunflower seeds, walnuts, pistachios, etc.)	0.0471
10.	Whole-grain foods (corn, millet, black rice, buckwheat, sorghum, etc.)	0.0564
11.	Sweets, cakes, desserts, sugary beverages (juice, sweet tea), honey, etc.*	0.0927
12.	Deep-fried foods (fried meatballs, deep-fried dough sticks, oil cakes, etc.) *	0.0803
13.	High-salt foods (pickles, salted duck eggs, sauces, etc.) *	0.0890
14.	Spicy / pungent foods (chili peppers; not onions, garlic, or ginger) *	0.0509
15.	What is your main staple food? (1)Coarse grains (corn, millet, buckwheat, etc.), (2)Refined grains (rice, wheat flour, etc.)	0.0611

16.	Which type of oil is most often used for cooking in your household? ⑴Vegetable oil (peanut, soybean, corn, etc.), ⑵Animal fat (lard, etc.)	0.0559
